# P-1229. Characterization of Ampicillin (AMP) and Vancomycin (VAN) Pharmacokinetics-Pharmacodynamics (PK-PD) in a Neutropenic Murine Invasive Enterococcal Infection Model

**DOI:** 10.1093/ofid/ofaf695.1421

**Published:** 2026-01-11

**Authors:** Brian D VanScoy, Allison N Seyfried, Alexander S MacGregor, Micah Nasman, Christopher M Rubino, Rodrigo E Mendes, Helio Sader, Paul G Ambrose, Sujata M Bhavnani

**Affiliations:** Institute for Clinical Pharmacodynamics, Schenectady, NY; Institute for Clinical Pharmacodynamics, Inc., Schenectady, New York; Institute for Clinical Pharmacodynamics, Inc., Schenectady, New York; Institute for Clinical Pharmacodynamics, Inc., Schenectady, New York; Institute for Clinical Pharmacodynamics, Schenectady, NY; Element Iowa City (JMI Laboratories), North Liberty, IA; Institute for Clinical Pharmacodynamics, Schenectady, NY; Institute for Clinical Pharmacodynamics, Schenectady, NY

## Abstract

**Background:**

AMP and VAN were developed in the 1950s and have remained important therapies for enterococcal infections. Current treatment decisions are based on *in vitro* susceptibility test interpretive criteria (STIC) using epidemiology data rather than robust PK-PD data due to the poor growth of *Enterococcus* species in standard pre-clinical infection models. The goal of these studies was to characterize the PK-PD of AMP and VAN against panels of *Enterococcus faecalis* and *E. faecium* isolates using a neutropenic murine invasive enterococcal infection model that we developed.

**Figure:**
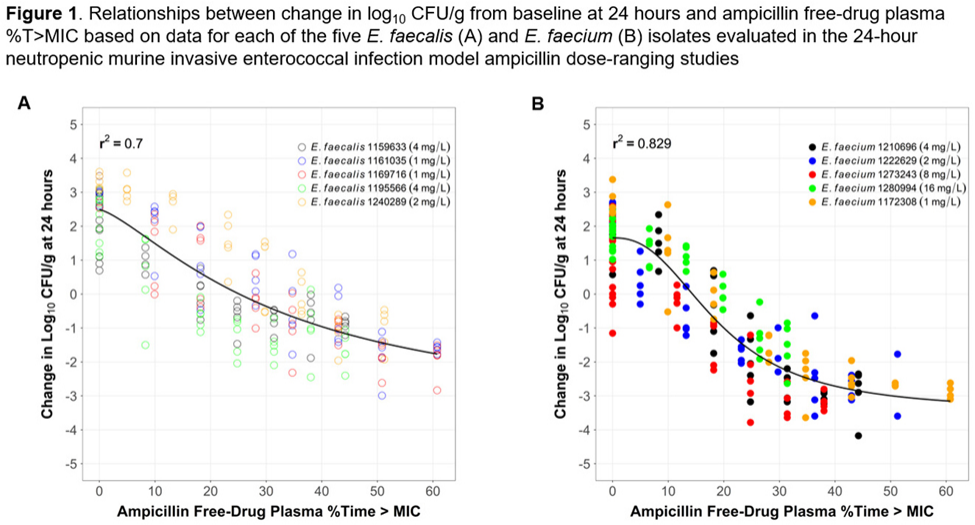

**Figure:**
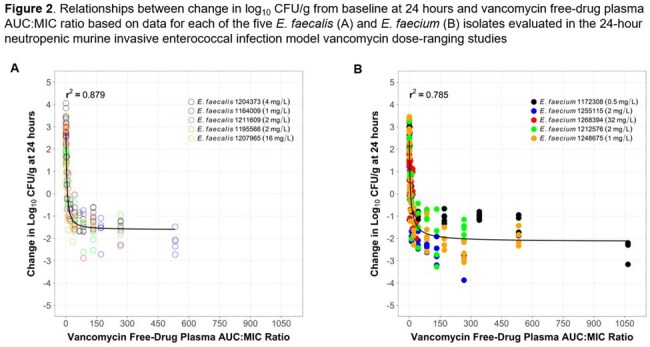

**Methods:**

Single-dose PK studies were completed in neutropenic mice infected with *E. faecalis* ATCC 29212. Plasma samples were collected over 7 time points following subcutaneous administration of 4 doses of AMP (4.50, 37.5, 150, and 600 mg/kg) or VAN (0.35, 7.00, 64.0, and 200 mg/kg). 24-hour dose-ranging studies were completed using 5 isolates of each of *E. faecium* and *E. faecali*s exposed to 7 different AMP or VAN dosing regimens administered every 6 hours. Population PK models were used to determine AMP free-drug plasma percent time above MIC (*f* %T >MIC) and VAN free-drug plasma area under the concentration-time curve over 24 hours (*f*AUC) values for these doses. Relationships between change in log_10_ CFU/g from baseline at 24 hours and each respective PK-PD index were fit using Hill-type models to determine the magnitude of the index required to achieve net bacterial stasis and a 1-log_10_ CFU/g reduction from baseline at 24 hours for each isolate and pooled by species.

**Results:**

Relationships between change in log_10_ CFU/g and each of AMP *f* %T >MIC and VAN *f*AUC:MIC ratio described the data well for *E. faecalis* and *E. faecium* (Figures 1 and 2). Median AMP *f* %T >MIC targets associated with net bacterial stasis and a 1-log_10_ CFU reduction from baseline were 25.5 and 44.7%, respectively, for *E. faecalis*, and 16.5 and 20.3%, respectively, for *E. faecium*. Median VAN *f*AUC:MIC ratio targets associated with these endpoints were 9.61 and 25.5, respectively, for *E. faecalis*, and 6.62 and 18.3, respectively, for *E. faecium.*

**Conclusion:**

Data from these neutropenic murine invasive enterococcal infection model studies allowed for the PK-PD characterization of AMP and VAN against *E. faecalis* and *E. faecium*, the PK-PD targets for which will support STIC evaluations.

**Disclosures:**

Brian D. VanScoy, B.S., A&G Pharma: Grant/Research Support|AiCuris Anti-infective Cures AG: Grant/Research Support|Albany College of Pharmacy and Health Sciences: Grant/Research Support|Albany Medical College: Grant/Research Support|AN2 Therapeutics: Grant/Research Support|Antabio SAS: Grant/Research Support|Apogee Biologics, Inc.: Grant/Research Support|Arcutis Biotherapeutics, Inc.: Grant/Research Support|B. Braun Medical, Inc.: Grant/Research Support|Basilea Pharmaceutica: Grant/Research Support|Cumberland Pharmaceuticals, Inc.: Grant/Research Support|Debiopharm: Grant/Research Support|Elion Therapeutics, Inc.: Grant/Research Support|Entasis Therapeutics: Grant/Research Support|Excalibur Pharmaceuticals, Inc.: Grant/Research Support|Fedora Pharmaceuticals: Grant/Research Support|Genentech: Grant/Research Support|Global Antibiotic Research & Development Partnership: Grant/Research Support|Inotrem: Grant/Research Support|Insmed, Inc.: Grant/Research Support|Institute for Clinical Pharmacodynamics, Inc.: Employee|Invivyd, Inc.: Grant/Research Support|Iterum Therapeutics Limited: Grant/Research Support|Kaizen Bioscience: Grant/Research Support|Lassen Therapeutics, Inc.: Grant/Research Support|Matinas Biopharma: Grant/Research Support|Meiji Seika Pharma Co., Ltd.: Grant/Research Support|Melinta Therapeutics: Grant/Research Support|Nabriva Therapeutics AG: Grant/Research Support|National Institutes of Health: Grant/Research Support|Novobiotic Pharmaceuticals LLC.: Grant/Research Support|Paratek Pharmaceuticals, Inc.: Grant/Research Support|Pfizer, Inc.: Grant/Research Support|Praxis Precision Medicines, Inc.: Grant/Research Support|PTC Therapeutics: Grant/Research Support|PureTech LYT 100, Inc.: Grant/Research Support|Qpex Biopharma: Grant/Research Support|Renibus Therapeutics: Grant/Research Support|Sagimet Biosciences, Inc.: Grant/Research Support|Schrodinger, Inc.: Grant/Research Support|Sfunga Therapeutics: Grant/Research Support|Shionogi, Inc.: Grant/Research Support|Spero Therapeutics: Grant/Research Support|Spruce Biosciences, Inc.: Grant/Research Support|UCB Biosciences, Inc.: Grant/Research Support|United States Food and Drug Administration: FDA Contract Number: 75F40123C00140|University of Wisconsin: Grant/Research Support|UT Southwestern: Grant/Research Support|VenatoRx Pharmaceuticals, Inc.: Grant/Research Support|Wockhardt Bio AG: Grant/Research Support|Zogenix International: Grant/Research Support Allison N. Seyfried, M.S., A&G Pharma: Grant/Research Support|AiCuris Anti-infective Cures AG: Grant/Research Support|Albany College of Pharmacy and Health Sciences: Grant/Research Support|Albany Medical College: Grant/Research Support|AN2 Therapeutics: Grant/Research Support|Antabio SAS: Grant/Research Support|Apogee Biologics, Inc.: Grant/Research Support|Arcutis Biotherapeutics, Inc.: Grant/Research Support|B. Braun Medical, Inc.: Grant/Research Support|Basilea Pharmaceutica: Grant/Research Support|Cumberland Pharmaceuticals, Inc.: Grant/Research Support|Debiopharm: Grant/Research Support|Elion Therapeutics, Inc.: Grant/Research Support|Entasis Therapeutics, Inc.: Grant/Research Support|Excalibur Pharmaceuticals, Inc.: Grant/Research Support|Fedora Pharmaceuticals: Grant/Research Support|Genentech: Grant/Research Support|Global Antibiotic Research & Development Partnership: Grant/Research Support|Inotrem: Grant/Research Support|Insmed, Inc.: Grant/Research Support|Institute for Clinical Pharmacodynamics, Inc.: Employee|Invivyd, Inc.: Grant/Research Support|Iterum Therapeutics Limited: Grant/Research Support|Kaizen Bioscience: Grant/Research Support|Lassen Therapeutics, Inc.: Grant/Research Support|Matinas Biopharma: Grant/Research Support|Meiji Seika Pharma Co., Ltd.: Grant/Research Support|Melinta Therapeutics: Grant/Research Support|Nabriva Therapeutics AG: Grant/Research Support|National Institutes of Health: Grant/Research Support|Novobiotic Pharmaceuticals LLC.: Grant/Research Support|Paratek Pharmaceuticals, Inc.: Grant/Research Support|Pfizer, Inc.: Grant/Research Support|Praxis Precision Medicines, Inc.: Grant/Research Support|PTC Therapeutics: Grant/Research Support|PureTech LYT 100, Inc.: Grant/Research Support|Qpex Biopharma: Grant/Research Support|Renibus Therapeutics: Grant/Research Support|Sagimet Biosciences, Inc.: Grant/Research Support|Schrodinger, Inc.: Grant/Research Support|Sfunga Therapeutics: Grant/Research Support|Shionogi, Inc.: Grant/Research Support|Spero Therapeutics: Grant/Research Support|Spruce Biosciences, Inc.: Grant/Research Support|UCB Biosciences, Inc.: Grant/Research Support|United States Food and Drug Administration: FDA Contract Number: 75F40123C00140|University of Wisconsin: Grant/Research Support|UT Southwestern: Grant/Research Support|VenatoRx Pharmaceuticals, Inc.: Grant/Research Support|Wockhardt Bio AG: Grant/Research Support|Zogenix International: Grant/Research Support Alexander S. MacGregor, B.S., A&G Pharma: Grant/Research Support|AiCuris Anti-infective Cures AG: Grant/Research Support|Albany College of Pharmacy and Health Sciences: Grant/Research Support|Albany Medical College: Grant/Research Support|AN2 Therapeutics: Grant/Research Support|Antabio SAS: Grant/Research Support|Apogee Biologics, Inc.: Grant/Research Support|Arcutis Biotherapeutics, Inc.: Grant/Research Support|B. Braun Medical, Inc.: Grant/Research Support|Basilea Pharmaceutica: Grant/Research Support|Cumberland Pharmaceuticals, Inc.: Grant/Research Support|Debiopharm: Grant/Research Support|Elion Therapeutics, Inc.: Grant/Research Support|Entasis Therapeutics: Grant/Research Support|Excalibur Pharmaceuticals, Inc.: Grant/Research Support|Fedora Pharmaceuticals: Grant/Research Support|Genentech: Grant/Research Support|Global Antibiotic Research & Development Partnership: Grant/Research Support|Inotrem: Grant/Research Support|Insmed, Inc.: Grant/Research Support|Institute for Clinical Pharmacodynamics, Inc.: Employee|Invivyd, Inc.: Grant/Research Support|Iterum Therapeutics Limited: Grant/Research Support|Kaizen Bioscience: Grant/Research Support|Lassen Therapeutics, Inc.: Grant/Research Support|Matinas Biopharma: Grant/Research Support|Meiji Seika Pharma Co., Ltd.: Grant/Research Support|Melinta Therapeutics: Grant/Research Support|Nabriva Therapeutics AG: Grant/Research Support|National Institutes of Health: Grant/Research Support|Novobiotic Pharmaceuticals LLC.: Grant/Research Support|Paratek Pharmaceuticals, Inc.: Grant/Research Support|Pfizer, Inc.: Grant/Research Support|Praxis Precision Medicines, Inc.: Grant/Research Support|PTC Therapeutics: Grant/Research Support|PureTech LYT 100, Inc.: Grant/Research Support|Qpex Biopharma: Grant/Research Support|Renibus Therapeutics: Grant/Research Support|Sagimet Biosciences, Inc.: Grant/Research Support|Schrodinger, Inc.: Grant/Research Support|Sfunga Therapeutics: Grant/Research Support|Shionogi, Inc.: Grant/Research Support|Spero Therapeutics: Grant/Research Support|Spruce Biosciences, Inc.: Grant/Research Support|UCB Biosciences, Inc.: Grant/Research Support|United States Food and Drug Administration: FDA Contract Number: 75F40123C00140|University of Wisconsin: Grant/Research Support|UT Southwestern: Grant/Research Support|VenatoRx Pharmaceuticals, Inc.: Grant/Research Support|Wockhardt Bio AG: Grant/Research Support|Zogenix International: Grant/Research Support Micah Nasman, B.S., A&G Pharma: Grant/Research Support|AiCuris Anti-infective Cures AG: Grant/Research Support|Albany College of Pharmacy and Health Sciences: Grant/Research Support|Albany Medical College: Grant/Research Support|AN2 Therapeutics: Grant/Research Support|Antabio SAS: Grant/Research Support|Apogee Biologics, Inc.: Grant/Research Support|Arcutis Biotherapeutics, Inc.: Grant/Research Support|B. Braun Medical, Inc.: Grant/Research Support|Basilea Pharmaceutica: Grant/Research Support|Cumberland Pharmaceuticals, Inc.: Grant/Research Support|Debiopharm: Grant/Research Support|Elion Therapeutics, Inc.: Grant/Research Support|Entasis Therapeutics: Grant/Research Support|Excalibur Pharmaceuticals, Inc.: Grant/Research Support|Fedora Pharmaceuticals: Grant/Research Support|Genentech: Grant/Research Support|Global Antibiotic Research & Development Partnership: Grant/Research Support|Inotrem: Grant/Research Support|Insmed, Inc.: Grant/Research Support|Institute for Clinical Pharmacodynamics, Inc.: Employee|Invivyd, Inc.: Grant/Research Support|Iterum Therapeutics Limited: Grant/Research Support|Kaizen Bioscience: Grant/Research Support|Lassen Therapeutics, Inc.: Grant/Research Support|Matinas Biopharma: Grant/Research Support|Meiji Seika Pharma Co., Ltd.: Grant/Research Support|Melinta Therapeutics: Grant/Research Support|Nabriva Therapeutics AG: Grant/Research Support|National Institutes of Health: Grant/Research Support|Novobiotic Pharmaceuticals LLC.: Grant/Research Support|Paratek Pharmaceuticals, Inc.: Grant/Research Support|Pfizer, Inc.: Grant/Research Support|Praxis Precision Medicines, Inc.: Grant/Research Support|PTC Therapeutics: Grant/Research Support|PureTech LYT 100, Inc.: Grant/Research Support|Qpex Biopharma: Grant/Research Support|Renibus Therapeutics: Grant/Research Support|Sagimet Biosciences, Inc.: Grant/Research Support|Schrodinger, Inc.: Grant/Research Support|Sfunga Therapeutics: Grant/Research Support|Shionogi, Inc.: Grant/Research Support|Spero Therapeutics: Grant/Research Support|Spruce Biosciences, Inc.: Grant/Research Support|UCB Biosciences, Inc.: Grant/Research Support|United States Food and Drug Administration: FDA Contract Number: 75F40123C00140|University of Wisconsin: Grant/Research Support|UT Southwestern: Grant/Research Support|VenatoRx Pharmaceuticals, Inc.: Grant/Research Support|Wockhardt Bio AG: Grant/Research Support|Zogenix International: Grant/Research Support Christopher M. Rubino, PharmD, A&G Pharma: Grant/Research Support|AiCuris Anti-infective Cures AG: Grant/Research Support|Albany College of Pharmacy and Health Sciences,: Grant/Research Support|Albany Medical College: Grant/Research Support|AN2 Therapeutics: Grant/Research Support|Antabio SAS: Grant/Research Support|Apogee Biologics, Inc.: Grant/Research Support|Arcutis Biotherapeutics, Inc.: Grant/Research Support|B. Braun Medical, Inc.: Grant/Research Support|Basilea Pharmaceutica: Grant/Research Support|Cumberland Pharmaceuticals, Inc.: Grant/Research Support|Debiopharm: Grant/Research Support|Elion Therapeutics, Inc.: Grant/Research Support|Entasis Therapeutics: Grant/Research Support|Excalibur Pharmaceuticals, Inc.: Grant/Research Support|Fedora Pharmaceuticals: Grant/Research Support|Genentech: Grant/Research Support|Global Antibiotic Research & Development Partnership: Grant/Research Support|Inotrem: Grant/Research Support|Insmed, Inc.: Grant/Research Support|Institute for Clinical Pharmacodynamics, Inc.: Ownership Interest|Invivyd, Inc.: Grant/Research Support|Iterum Therapeutics Limited: Grant/Research Support|Kaizen Bioscience: Grant/Research Support|Lassen Therapeutics, Inc.: Grant/Research Support|Matinas Biopharma: Grant/Research Support|Meiji Seika Pharma Co., Ltd.: Grant/Research Support|Melinta Therapeutics: Grant/Research Support|Nabriva Therapeutics AG: Grant/Research Support|National Institutes of Health: Grant/Research Support|Novobiotic Pharmaceuticals LLC.: Grant/Research Support|Paratek Pharmaceuticals, Inc.: Grant/Research Support|Pfizer, Inc.: Grant/Research Support|Praxis Precision Medicines, Inc.: Grant/Research Support|pRxcision, Inc.: Ownership Interest|PTC Therapeutics: Grant/Research Support|PureTech LYT 100, Inc.: Grant/Research Support|Qpex Biopharma: Grant/Research Support|Renibus Therapeutics: Grant/Research Support|Sagimet Biosciences, Inc.: Grant/Research Support|Schrodinger, Inc.: Grant/Research Support|Sfunga Therapeutics: Grant/Research Support|Shionogi, Inc.: Grant/Research Support|Spero Therapeutics: Grant/Research Support|Spruce Biosciences, Inc.: Grant/Research Support|UCB Biosciences, Inc.: Grant/Research Support|United States Food and Drug Administration: FDA Contract Number: 75F40123C00140|University of Wisconsin: Grant/Research Support|UT Southwestern: Grant/Research Support|VenatoRx Pharmaceuticals, Inc.: Grant/Research Support|Wockhardt Bio AG: Grant/Research Support|Zogenix International: Grant/Research Support Rodrigo E. Mendes, PhD, GSK: Grant/Research Support|Shionogi & Co., Ltd.: Grant/Research Support|United States Food and Drug Administration: FDA Contract Number: 75F40123C00140 Helio Sader, United States Food and Drug Administration: FDA Contract Number: 75F40123C00140 Paul G. Ambrose, PharmD; MS; FIDSA, A&G Pharma: Grant/Research Support|AiCuris Anti-infective Cures AG: Grant/Research Support|Albany College of Pharmacy and Health Sciences: Grant/Research Support|Albany Medical College: Grant/Research Support|AN2 Therapeutics: Grant/Research Support|Antabio SAS: Grant/Research Support|Apogee Biologics, Inc.: Grant/Research Support|Arcutis Biotherapeutics, Inc.: Grant/Research Support|B. Braun Medical, Inc.: Grant/Research Support|Basilea Pharmaceutica: Grant/Research Support|Cumberland Pharmaceuticals, Inc.: Grant/Research Support|Debiopharm: Grant/Research Support|Elion Therapeutics, Inc.: Grant/Research Support|Entasis Therapeutics: Grant/Research Support|Excalibur Pharmaceuticals, Inc.: Grant/Research Support|Fedora Pharmaceuticals: Grant/Research Support|Genentech: Grant/Research Support|GlaxoSmithKline: Advisor/Consultant|Global Antibiotic Research & Development Partnership: Grant/Research Support|Inotrem: Grant/Research Support|Insmed, Inc.: Grant/Research Support|Institute for Clinical Pharmacodynamics, Inc.: Ownership Interest|Invivyd, Inc.: Grant/Research Support|Iterum Therapeutics Limited: Grant/Research Support|Kaizen Bioscience: Grant/Research Support|Lassen Therapeutics, Inc.: Grant/Research Support|Matinas Biopharma: Grant/Research Support|Meiji Seika Pharma Co., Ltd.: Grant/Research Support|Melinta Therapeutics: Grant/Research Support|Nabriva Therapeutics AG: Grant/Research Support|National Institutes of Health: Grant/Research Support|Novobiotic Pharmaceuticals LLC.: Grant/Research Support|Paratek Pharmaceuticals, Inc.: Grant/Research Support|Pfizer, Inc.: Grant/Research Support|Praxis Precision Medicines, Inc.: Grant/Research Support|pRxcision, Inc.: Ownership Interest|PTC Therapeutics: Grant/Research Support|PureTech LYT 100, Inc.: Grant/Research Support|Qpex Biopharma: Grant/Research Support|Renibus Therapeutics: Grant/Research Support|Sagimet Biosciences, Inc.: Grant/Research Support|Schrodinger, Inc.: Grant/Research Support|Sfunga Therapeutics: Grant/Research Support|Shionogi, Inc.: Advisor/Consultant|Shionogi, Inc.: Grant/Research Support|Spero Therapeutics: Grant/Research Support|Spruce Biosciences, Inc.: Grant/Research Support|UCB Biosciences, Inc.: Grant/Research Support|United States Food and Drug Administration: FDA Contract Number: 75F40123C00140|University of Wisconsin: Grant/Research Support|UT Southwestern: Grant/Research Support|VenatoRx Pharmaceuticals, Inc.: Grant/Research Support|Wockhardt Bio AG: Grant/Research Support|Zogenix International: Grant/Research Support Sujata M. Bhavnani, PharmD; MS; FIDSA, A&G Pharma: Grant/Research Support|AiCuris Anti-infective Cures AG: Grant/Research Support|Albany College of Pharmacy and Health Sciences: Grant/Research Support|Albany Medical College: Grant/Research Support|AN2 Therapeutics: Grant/Research Support|Antabio SAS: Grant/Research Support|Apogee Biologics, Inc.: Grant/Research Support|Arcutis Biotherapeutics, Inc.: Grant/Research Support|Aurobac Therapeutics: Advisor/Consultant|B. Braun Medical, Inc.: Grant/Research Support|Basilea Pharmaceutica: Grant/Research Support|Cumberland Pharmaceuticals, Inc.: Grant/Research Support|Debiopharm: Grant/Research Support|Elion Therapeutics, Inc.: Grant/Research Support|Entasis Therapeutics: Grant/Research Support|Excalibur Pharmaceuticals, Inc: Grant/Research Support|Fedora Pharmaceuticals: Grant/Research Support|Genentech: Grant/Research Support|GlaxoSmithKline: Advisor/Consultant|Global Antibiotic Research & Development Partnership: Grant/Research Support|Inotrem: Grant/Research Support|Insmed, Inc.: Grant/Research Support|Institute for Clinical Pharmacodynamics: Ownership Interest|Invivyd, Inc.: Grant/Research Support|Iterum Therapeutics Limited: Grant/Research Support|Kaizen Bioscience: Grant/Research Support|Lassen Therapeutics, Inc.: Grant/Research Support|Matinas Biopharma: Grant/Research Support|Meiji Seika Pharma Co., Ltd.: Grant/Research Support|Melinta Therapeutics: Grant/Research Support|Nabriva Therapeutics AG: Grant/Research Support|National Institutes of Health: Grant/Research Support|Novobiotic Pharmaceuticals LLC.: Grant/Research Support|Paratek Pharmaceuticals, Inc.: Grant/Research Support|Pfizer, Inc.: Grant/Research Support|Praxis Precision Medicines, Inc.: Grant/Research Support|pRxcision, Inc.: Ownership Interest|PTC Therapeutics: Grant/Research Support|PureTech LYT 100, Inc.: Grant/Research Support|Qpex Biopharma: Grant/Research Support|Renibus Therapeutics: Grant/Research Support|Sagimet Biosciences, Inc.: Grant/Research Support|Schrodinger, Inc.: Grant/Research Support|Sfunga Therapeutics: Grant/Research Support|Shionogi, Inc.: Advisor/Consultant|Shionogi, Inc.: Grant/Research Support|Spero Therapeutics: Grant/Research Support|Spruce Biosciences, Inc.: Grant/Research Support|UCB Biosciences, Inc.: Grant/Research Support|United States Food and Drug Administration: FDA Contract Number: 75F40123C00140|University of Wisconsin: Grant/Research Support|UT Southwestern: Grant/Research Support|VenatoRx Pharmaceuticals, Inc.: Grant/Research Support|Wockhardt Bio AG: Grant/Research Support|Zogenix International: Grant/Research Support

